# Supplementary material for: Long-lasting insecticidal nets (LLINs) use among household members for protection against mosquito bite in Mogadishu districts
Source: PLOS Glob Public Health. 2023 Mar 16;3(3):e0000724. doi: 10.1371/journal.pgph.0000724 (PMC10019640; doi:10.1371/journal.pgph.0000724)
Supplement: Attachment — Submitted filename: Responders to reviewers et al malaria.docx [file pgph.0000724.s002.docx]

***Response to comments from the handling editor***

Journal Requirements:

1. **Please amend your ethics statement stating that the IRB specifically approved your study.**

*We have now amended the ethics statement as follows: ‘This study was reviewed and approved by Somali Medical Association (SMA).’*

2. **Please update your online Competing Interests statement. If you have no competing interests to declare, please state: “The authors have declared that no competing interests exist.”**

*As advised, we have indicated that* “*The authors have declared that no competing interests exist.” Under the Competing Interest subs-heading in the manuscript.*

3. **We do not publish any copyright or trademark symbols that usually accompany proprietary names, eg (R), (C), or TM  (e.g. next to drug or reagent names). Please remove all instances of trademark/copyright symbols throughout the text, including ® (Olyset®) on page 19.**

*Thank a lot. We have since removed in the “®” in “Olyset®” in the reference as sufficiently directed. The reference now reads: “Mejía P, Teklehaimanot HD, Tesfaye Y, Teklehaimanot A. Physical condition of Olyset nets after five years of utilization in rural western Kenya. Malar J 2013;12:1–11.* [*https://doi.org/10.1186/1475-2875-12-158*](https://doi.org/10.1186/1475-2875-12-158)*.” We have also removed “^TM^” in the “Excel^TM^.”*

**Reviewer #1: Thank you to the authors for contributing to the understanding of LLIN ownership and use in Somalia. The authors present that though ownership in Somalia is low, net use among households that do own nets is high. This shows promising evidence that increasing net access and ownership may likely lead to increases in net use at the household level. Please see my comments below to explain the review questions and to address other minor issues:**

**1. I responded "partly" to the question regarding meeting publication criteria. I believe the manuscript meets the criteria, however some clarification on the ethical clearance is needed. In the manuscript, ethical considerations section, the authors state "the study was presented for review" to the institutional review board. Please provide clarification on whether the study was approved through the IRB. (As it reads, it may be interpreted that the IRB simply received the application, but does not state the decision of that review)**

*This has been corrected. We have amended the ethics statement read as follows: ‘This study was reviewed and approved by Somali Medical Association (SMA).’*

**2. Statistical analyses. It appears the analyses have been conducted correctly. However, for replicability, more information is needed for the reader. In describing the Generalized Estimating Equations, please report what parameters were used (family, identity link function, correlation structure, and type of standard errors). For an example of GEEs used in related malaria literature, see Santos et al 2019,** [**https://malariajournal.biomedcentral.com/articles/10.1186/s12936-019-2908-6**](https://malariajournal.biomedcentral.com/articles/10.1186/s12936-019-2908-6)
*We have now amended and included an exchangeable correlation structure and binomial family with a logit link (the default link function).*

**3. PLOS Data Policy. I responded No- however, the authors partially meet this requirement. The authors provided a Google Spreadsheet with the raw data. The only reason I state this does not meet the PLOS data policy is because the spreadsheet requires a password and only the corresponding author's contact information is provided to help provide access. Within the Data Policy statement, PLOS does not allow for only a single author to be the contact point for data access. Please review the policy and find an alternative way to allow for password access to the dataset.**

*We apologise for this. We have since reviewed the policy and now provide an easy to access data in an Excel sheet in accordance with the PLOS Data Policy.*

**Other Comments:

1. The objective of this manuscript is to measure which factors are associated with net use among households. It was found that net ownership was low (155 households own nets, or 37% of households). This reduces the sample size to 155, which likely affects the power of the GEE models to detect significant associations. Among those 155 households, net use was rather high (199 nets out of 237 total nets were used the prior night=84% use). Realizing the objective of the manuscript is to measure factors association with use- it would seem that more exploration of factors associated with net ownership would address the larger issue here. If the authors have any data on factors related to net ownership, I highly recommend incorporating this into the manuscript.**

*We can confirm that we have data on net ownership and factors likely to likely related to it. As you have recommended, we have included a Table for factors associated with net ownership (please see Table 3 of this manuscript).*

**2. Section 4.4.1- The authors state that there were 2 nets per household among the households that owned nets. However, 237 total nets/155 households that own nets = 1.53 nets per household. Please correct this statement.**

*These changes had been made for the present revised version and further amended to indicate “The median number of nets per household was 2 (interquartile range: 1.0, 2.0) nets in this study” in the results section (page 13, paragraph 1). We would also like to report that our data for this variable was non-normal and therefore we chose to use the median instead of the mean. However, if there is justification to use mean as suggested, we are happy to do so.*

**3. In the title, I recommend using the term "****insecticidal" to remain consistent with the field and the terminology used in the abstract. This will allow for the manuscript to be found during systematic reviews and literature searches in the future.**

*Thanks a lot for this. We have now amended the title as follows to: ‘Long-lasting insecticidal nets (LLINS) use among household members for protection against mosquito bite in Mogadishu districts’*

**4. Was data collected during the transmission season, or does transmission occur year-round? Please clarify the Plasmodium transmission season in the study area in relation to when data were collected (this could impact ownership and use rates)**

*We collected data between 1^st^ November 2020 and 31^st^ March 2021. “Malaria transmission in Somalia varies from hypoendemic to mesoendemic with areas having areas of year-round transmission with two peaks of an increased number of cases during the two wet seasons: April to June and October to November.”*^[1]^

**5. On page 10, the authors state “In cases where there was only one net, the net was majorly used by children under 5 years old (37.3%) however, difference in frequency was statistically attenuated (*not significant – in significant* ) (p=0.190)." Please explain what this statement means. It appears (based on the p-value), that there was no statistical difference in age category of net use. Please clarify.**

*Apologies for the lack of clarity and vagueness on this. We have since amended this as “In cases where there was only one net, the net was majorly used by children under five years old (37.3%) followed by children under five (not infants) and pregnant women, however, the difference in frequency by use of the net the previous night was not statistically significant (p=0.190).”*

**6. In the Introduction, the authors state "However, a dramatic reduction in malaria infection rates has also been shown". Please further explain this sentence. Does this refer to Somalia or a broader geographic context? In what time period, and by how much?**

*We have now added a reference from World Health Organization (2021) for clarification of this. “Published malariometric surveys in Mogadishu districts (Benadir region) have shown prevalence as high as 50% [4] (Malaria Indicator Survey 2014) and estimates between 0% and 52% in the south. However, a dramatic reduction in malaria cases and death was seen in 2021 compared to previous years due to the malaria control program in the country [5].”*

**8. Methods section: the manuscript states that the target population is all household members in the region. However, the unit of analysis is the household level, so the target population should match the unit of analysis.**

*Thanks a lot. We have amended this as advised in the section.*

**Study design. This was a cross-sectional household study conducted using a stratified two-stage cluster survey design.
Authors should clarify the two stages e.g. enumeration areas and households**

*Thanks a lot. We have provided more information in the Sampling frame and sampling procedure sub-section.* *The sampling frame was a complete list of geographic information system spatially constructed (digitized) dwelling structures in the Mogadishu districts provided described by in the SHDS 2020 and UNFPA reports [53,54,56]. In the SHDS 2020, the urban areas have 50 – 49 dwelling structures within each of the statistical units (enumeration areas – EAs) as shown in Omar et al. [19].*

*We envisaged a two-stage stratified cluster sampling design, however, due to the COVID-19 pandemic, we adjusted to a three-stage cluster design. In the first sampling stage, we selected seven districts randomly out of a total of 18 Mogadishu districts. We then sampled 11 enumeration areas out of each of the seven districts in the second stage by probability proportional to the estimated size (PPS) of digitized dwelling structures described in the SHDS 2020. In the third stage, we systematically sampled eight households selected from each EA. In the first sampling stage, 11 EAs were selected in each district by random sampling by probability proportional to size (PPS) of digitized dwelling structures. In the second stage, systematic random sampling was used to 8 households in each EA.*

**Sample size. According to the 2014 Somalia Malaria Indicator Survey [4] , 20.1% of household members slept under an LLIN. The indicator above is based on household members whereas the sample size calculation was done for households. The authors should provide a justification for their approach.**

*Thanks a lot for this observation. In Somalia, there is paucity of epidemiological data – including recent data – from which to base sample size calculations, our plan was to assume a conservative estimate of net utilization of about 20% by household members but then we thought that taking this at the household would give us even sufficient data to account for the net ownership and net-level use at the household as well as individual household members use of the nets. However, due to the and the changing security scenarios in Mogadishu and the COVID-19 pandemic also necessitated shortening the questionnaire to collect only net-level use of LLIN as advised by ethical reviewers. We believe reached an adequate sample given that we reached 409 households with a total of 2442 members as well as obtained net-level use from a subset of 155 households owning nets with 942 household members.*

**An overarching comment is the lack of clarity as to whether the results are for households or household members. Since the authors note that all household members were surveyed, it is unclear how Table 1 on household characteristics and Table 2 on respondents are the same N of 409.**

*Apologies for this lack of clarity. In Table 1, we report the characteristics of the head of the household as well as the characteristics of the household as reported by the head of the household. We have amended the table title to read “Household head’s individual and household-level characteristics stratified by ownership of LLIN among households surveyed, Mogadishu – Somalia, 2021” which gives N=409 for all the households. In table 2, we report knowledge of malaria causes as reported by the head of the household/or the oldest adult found during the survey date. We have amended the table title to read “Table 2. Reported knowledge of causes and preventive measures and information about bed nets use among households as reported by the household heads in the Mogadishu districts, Somalia, 2021.”*

**Table 2.
The current guidance on malaria knowledge is to note the percent of respondents who correctly only state malaria as being caused by mosquito bites. The authors should note this proportion in the table.**

*Thanks for guidance. We have amended this to show that knowledge is based on correctly stating mosquito bites.*

**Table 4 is hard to understand because it is unclear whether the unit of analysis is the net or the household member. The authors should explain how the different levels of information were collected in the methods section and then present net level results- number of nets observed, source, age shape, color, appearance, net used the previous night. Etc.**

*Thank you. In Table 4 (now Table 5), we present data on the nets and their utilization, that is, whether a household member slept in the net the previous night prior to the survey. We have since provided more information in the Methods Section s advised.* *The use (or not) of a particular LLIN the previous night was the dependent variable. We have changed the Table column heading to “Net used previous night” to bring clarity.*

**The row titled number of nets is confusing and unclear. It would only make sense if the data presented was at the household level.

In addition, further clarification of what the authors intend to convey is needed for the row titled “People using net if there is only one net available in the household (n=100)”. Is n=100 referring to households? Also the numbers do not add up to 100. Recommend that the authors present their information in a clear and concise way that clearly shows the rationale for the specific results.**
*Thanks for this. We have since broken it doen to “Number of nets available (continuous)*

*Number of nets available (groups)”, and “Number of nets available (one vs. ≥2)”. We have added more information in the Methods section: “Additionally, we recorded the reasons not owning nets, the physical appearance, source of the nets and utilization LLIN of a particular net (net-level) by the household members the night prior to the survey. We also asked the respondents the categories of people that use the nets when there is only one net in the household. This was particularly useful to characterize the groups of the at-risk population, especially the infants and others generally below the age of five as well as the pregnant women who are susceptible to malaria in pregnancy and how this influences net ownership and utilization.” To help explain why we collected information on who uses nets when it is only one. We found cases of 100 single nets, hence the analysis was restricted to this subset.*

**It is strongly recommended that the authors do not include percentages or statistics already noted in the results section
Universal coverage is no longer typically measured at the household level but better assessed at the individual or household member level**

*We have since removed the percentages and figures and only retained those that are not from the analysis but from cited literature. Unfortunately, our analyses were based on the net-level use so, we are only able to report on net level.*

**“LLIN being expensive (42.0%) was a major barrier to ownership.” Authors should discuss this in relation to the most recent mass ITN distribution in the study area (2019 - 2020). This should be done for the follow up section that states “Majority of the LLINs were from free mass distribution (55.7%) and market/retail shop (19.5%). This means that the local community are reliant on national or community-level mass distribution.”**

*We have reorganised it as advised. Thanks a lot.*

**“However, knowledge of preventive methods was considerably moderate.” This sentence should be discussed in relation to ITN utilization as in the previous sentence. (SAME AS ABOVE).**

*We have reorganised this as well.*

**“This study found that children under 5 years (61.9% households), pregnant women and elders – in that order – were given priority in situations where there was only one LLIN in a household.“ This presumes that only one person slept under a net. Can the authors verify this? (EXPLAIN).**

*I have amednded this and provided an explaination and citatation and an average househould size reported by the Somali Health and Demographic survey (2020).*

1. NMCPs/MoH. (2016). *Guidelines for the Diagnosis and Treatment of Malaria in Somalia*. https://reliefweb.int/sites/reliefweb.int/files/resources/malaria_diagnosis_and_treatment_guidelines_180316.pdf
